# Bimatoprost, latanoprost, and tafluprost induce differential expression of matrix metalloproteinases and tissue inhibitor of metalloproteinases

**DOI:** 10.1186/s12886-016-0202-8

**Published:** 2016-03-08

**Authors:** Hiroshi Yamada, Masahiko Yoneda, Masahiko Gosho, Tomohiro Kato, Masahiro Zako

**Affiliations:** Department of Opthalmology, Aichi Medical University, Nagakute, Aichi 480-1195 Japan; Department of Biochemistry and Molecular Biology, School of Nursing and Health, Aichi Prefectural University, Nagakute, Aichi 463-8502 Japan; Department of Clinical Trial and Clinical Epidemiology, Faculty of Medicine, University of Tsukuba, Tsukuba, Ibaraki 305-8575 Japan

**Keywords:** Human non-pigmented ciliary epithelial cells, Matrix metalloproteinases, Prostaglandin analog, Tissue inhibitor of metalloproteinases

## Abstract

**Background:**

Differences in the increase in matrix metalloproteinase (MMP) and decrease in tissue inhibitor of metalloproteinase (TIMP) activity may contribute to the different characteristics observed clinically on decreased intraocular pressure in patients with glaucoma or ocular hypertension. The purpose of this study was to investigate differences in the expression profiles of MMPs and TIMPs induced by the prostaglandin analogs bimatoprost, latanoprost, and tafluprost in human non-pigmented ciliary epithelial cells (HNPCECs).

**Methods:**

HNPCECs were cultured for 24 h with 0, 10, 100, or 1000 μM of the free acid forms of bimatoprost, latanoprost, and tafluprost. We measured the expression levels of MMPs and TIMPs using real-time polymerase chain reaction, and compared the results. Enzyme activities of MMP-2 and −9 in conditioned media were measured by gelatin zymography.

**Results:**

All prostaglandin analogs we examined dose-dependently increased expression levels of MMP-1, −2, −3, −9, and −17, whereas expression levels of TIMP-1 and −2 decreased with increasing concentrations of each analog. Each prostaglandin analog induced different levels of increases in MMPs and decreases in TIMPs.

**Conclusions:**

Unique expression profiles of MMPs and TIMPs induced by bimatoprost, latanoprost, and tafluprost, as shown in HNPCECs, may contribute to clinically different effects on intraocular pressure decreases in patients with glaucoma or ocular hypertension.

## Background

Bimatoprost (amide prodrug of 17-phenyl-PGF_2α_), latanoprost (ester prodrug of PGF_2α_), and tafluprost (difluoroprostaglandin derivative of PGF_2α_) are prostaglandin analogs (PGAs) available for clinical use to lower patients’ intraocular pressure (IOP). Recently, PGAs were approved as a first-line treatment for glaucoma based on their efficacy in lowering IOP, lack of relevant systemic side effects, and requirement for once-daily dosing [1].

The mechanism by which PGAs reduce IOP has been well-studied and is believed to occur by enhancement of uveoscleral outflow due to the regulation of matrix metalloproteinases (MMPs) and resulting remodeling of the extracellular matrix [2–4]. Clinically, we often experience different effects from the different PGAs. Different receptor subtypes for PGAs were proposed, and different PGAs may lower IOP by different mechanisms of action through these different receptor subtypes [5, 6]. However, the precise mechanism through which these PGAs exhibit different effects in individual patients is still unclear. Previous studies reported differential expression of MMPs in the ciliary body or ciliary muscle after bimatoprost, latanoprost, or tafluprost treatment [2–4, 7]. The activity of these endopeptidases is inhibited by endogenous tissue inhibitor of metalloproteinases (TIMPs); thus, the balanced expression of MMPs and TIMPs is important for maintaining homeostasis of the extracellular matrix and for reducing IOP by these PGAs.

Human non-pigmented ciliary epithelial cells (HNPCECs), which comprise the blood-aqueous barrier (BAB), are involved in controlling IOP. HNPCECs secrete aqueous humor. A decrease in aqueous humor secretion occurs in association with uveitis, especially uveitis involving the ciliary body epithelium (iridocyclitis) [8]. We previously showed that TNF-α (tumor necrosis factor-α) promotes the induction of MMPs in HNPCECs, which degrade claudin-1 and occludin, major constituents of tight-junctions between HNPCECs [9]. We found significantly increased permeability of a monolayer of HNPCECs after MMP treatment. We hypothesized that the balance of MMPs and TIMPs that degrade the components of tight junctions in the BAB in HNPCECs modifies the production of aqueous humor, which consequently decreases aqueous secretion and IOP.

In the present study, we measured and compared the expression levels of MMPs and TIMPs in HNPCECs cultured with various concentrations of bimatoprost, latanoprost, or tafluprost. Differences in the increase in MMP and decrease in TIMP activity may contribute to the different characteristics observed clinically on decreased IOP in patients with glaucoma or ocular hypertension.

## Methods

### Cell culture

HNPCECs were prepared as described previously [9].

### Cell treatment

Free-acid forms of bimatoprost, latanoprost, or tafluprost (Cayman Chemical Ann Arbor, MI, USA) were prepared in dimethyl sulfoxide (DMSO) and diluted to experimental concentrations (10, 100, and 1000 μM) with serum-free medium and added to the HNPCECs. The cells were then incubated for 24 h.

### RNA isolation and cDNA synthesis

Total cellular RNA was isolated from the HNPCECs, and total RNA was used as a template for cDNA synthesis with random primers as described previously [9].

### qPCR

cDNA was synthesized from total RNA (50 ng) for each treatment group. The cDNA served as the template for the real-time quantitative polymerase chain reaction (qPCR) assays. The primer sequences for human MMP-1, −2, −3, and −9 were described previously [9]. The primer sequences were human MMP-17 forward (5′-CACCAAGTGGAACAAGAGGAACCT-3′) and reverse (5′-TGGTAGTACGGCCGCATGATGGAGTGTGCA-3′). The primer sequences for human TIMP-1, and −2 were described previously [9]. The qPCR gene expression results were normalized as described previously [9]. The qPCR reaction was carried out as described previously [9].

### Gelatin zymography

Gelatin zymography was performed and gelatinolytic band densities were quantified as described previously [9].

### Statistical analysis

Experiments were repeated four times. The results are presented as the mean ± standard deviation (SD). Statistical analyses were performed using an analysis of variance model with Bonferroni’s post hoc test for multiple comparisons. The value measured without PAGs was defined as a control. We used the SAS 9.4 software (SAS Institute, Cary, NC, USA) for all statistical analyses. Differences with *P*-values < 0.05 were considered significant.

## Results

**Fig. 1:**
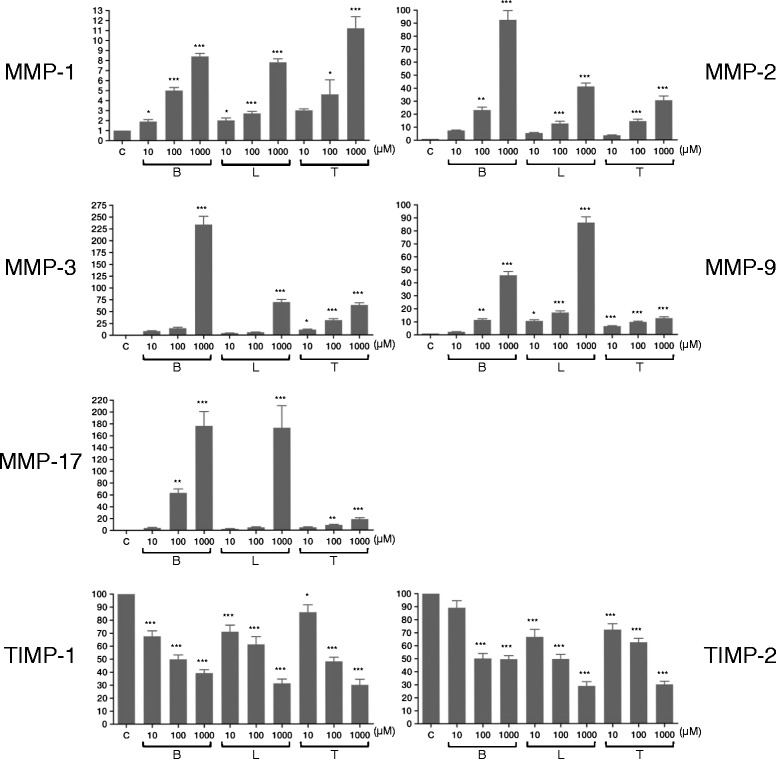
Quantitative PCR shows gene expression of matrix metalloproteinases (MMPs) and tissue inhibitor of metalloproteinases (TIMPs) in human non-pigmented ciliary epithelial cells (HNPCECs). MMP-1, MMP-2, MMP-3, MMP-9, and MMP-17 mRNA levels were up-regulated in the presence of increasing concentrations of each prostaglandin analog. TIMP-1 and TIMP-2 mRNA levels were down-regulated in the presence of increasing concentrations of each prostaglandin analog. Expression without prostaglandin analog was used as a control. B: bimatoprost, L: latanoprost, T: tafluprost. Data represent the mean ± SD (*n* = 4). *P < 0.05, **P < 0.01, ***P < 0.001 vs. control; one-way analysis of variance with Bonferroni’s correction

We examined the expression levels of MMPs and TIMPs in cellular extracts from HNPCECs treated with 10, 100, or 1000 μM of bimatoprost, latanoprost, or tafluprost. The qPCR results demonstrated that MMP-1, MMP-2, MMP-3, MMP-9, and MMP-17 mRNA expression levels were dose dependently increased with increasing bimatoprost, latanoprost, or tafluprost concentrations (Fig. 1, Table 1). When the concentrations of the PGAs were 1000 μM, the MMP-1, MMP-2, MMP-3, MMP-9, and MMP-17 expression levels significantly increased compared to the control levels. When the PGA concentration was 100 and 1000 μM, TIMP-1 and TIMP-2 mRNA expression levels in HNPCECs significantly decreased compared to the control levels.

**Table 1:** Comparison with control group

Response	Drug	Dose	Mean	SD	*P*
MMP-1	B	10	1.9	0.4	0.044
	B	100	5.0	0.6	<0.001
	B	1000	8.4	0.6	<0.001
	L	10	2.0	0.5	0.018
	L	100	2.7	0.4	<0.001
	L	1000	7.8	0.7	<0.001
	T	10	3.0	0.3	0.273
	T	100	4.6	2.9	0.023
	T	1000	11.2	2.3	<0.001
MMP-2	B	10	7.5	0.6	0.501
	B	100	23.1	4.5	0.002
	B	1000	92.5	14.2	<0.001
	L	10	5.5	0.6	0.100
	L	100	12.7	3.4	<0.001
	L	1000	41.2	5.2	<0.001
	T	10	3.6	0.6	0.706
	T	100	14.4	3.0	<0.001
	T	1000	30.5	6.4	<0.001
MMP-3	B	10	8.9	0.9	1.000
	B	100	14.9	3.8	0.604
	B	1000	234.0	34.7	<0.001
	L	10	4.2	0.8	1.000
	L	100	6.1	0.7	0.432
	L	1000	69.8	10.9	<0.001
	T	10	11.4	2.2	0.030
	T	100	31.6	5.8	<0.001
	T	1000	63.3	9.2	<0.001
MMP-9	B	10	2.3	0.3	1.000
	B	100	11.5	1.6	0.001
	B	1000	45.8	5.7	<0.001
	L	10	10.6	1.9	0.019
	L	100	17.0	2.5	<0.001
	L	1000	86.2	8.9	<0.001
	T	10	6.5	0.6	<0.001
	T	100	9.8	0.9	<0.001
	T	1000	12.6	2.1	<0.001
MMP-17	B	10	3.9	2.2	1.000
	B	100	63.2	13.4	0.006
	B	1000	176.5	47.9	<0.001
	L	10	2.3	1.1	1.000
	L	100	4.9	1.7	1.000
	L	1000	173.1	74.7	<0.001
	T	10	4.8	1.7	0.105
	T	100	8.8	1.7	0.002
	T	1000	18.5	4.8	<0.001
TIMP-1	B	10	67.6	8.3	<0.001
	B	100	49.8	6.7	<0.001
	B	1000	39.2	5.2	<0.001
	L	10	71.0	10.2	<0.001
	L	100	61.3	11.9	<0.001
	L	1000	31.3	6.5	<0.001
	T	10	85.9	11.5	0.038
	T	100	48.1	6.3	<0.001
	T	1000	30.0	8.7	<0.001
TIMP-2	B	10	89.2	10.8	0.082
	B	100	50.2	7.5	<0.001
	B	1000	49.6	5.3	<0.001
	L	10	66.7	11.6	<0.001
	L	100	49.7	7.1	<0.001
	L	1000	29.0	6.6	<0.001
	T	10	72.2	9.1	<0.001
	T	100	62.4	6.2	<0.001
	T	1000	30.1	4.6	<0.001

*P* one-way ANOVA with Bonferroni’s post hoc test for multiple comparisons

*B* bimatoprost, *L* latanoprost, *T* tafluprost

Dose: μM

**Fig. 2:**
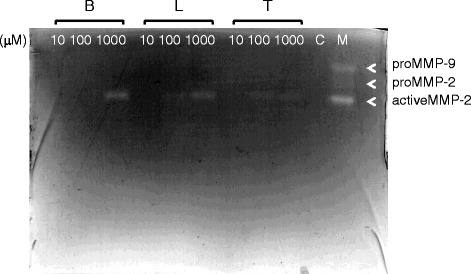
Gelatin zymogram showing the band densities of MMP-2 and MMP-9. Only bands of activeMMP-2 were detected after treatment with the prostaglandin analogs. No bands were detected without treatment (control lane). Samples containing 20 μg of protein were analyzed. B: bimatoprost, L: latanoprost, T: tafluprost. C: control, M: molecular weight marker. Molecular weights of proMMP-9, proMMP-2, and activeMMP-2 are 92 kDa, 72 kDa, and 62 kDa, respectively

We performed gelatin zymography to measure the enzymatic activity of MMP-2 and MMP-9 in HNPCECs treated with 10, 100, or 1000 μM bimatoprost, latanoprost, or tafluprost (Fig. 2). MMP-2 and MMP-9 controls were used to identify the location of each band (Fig. 2, arrowheads). We detected active MMP-2 forms, but not pro-MMP-9 or pro-MMP-2 forms. We performed a quantitative analysis of the band densities to evaluate active MMP-2 activity in HNPCECs treated with 10, 100, or 1000 μM of bimatoprost, latanoprost, or tafluprost. When the band density of active MMP-2 in HNPCECs treated with 1000 μM bimatoprost was defined as 100, the relative band densities of active MMP-2 in HNPCECs treated with 100 and 1000 μM latanoprost and tafluprost were calculated as 18 and 63 and 31 and 47, respectively. No band was detected in HNPCECs treated with 10 and 100 μM bimatoprost or 10 μM latanoprost and tafluprost. The relative mRNA expression levels of MMP-2 in HNPCECs treated with 1000 μM bimatoprost and with 100 or 1000 μM latanoprost or tafluprost were calculated as 100:14:45:16:33, respectively (Fig. 1), and this relationship appeared similar to the relative band densities of activeMMP-2 in HNPCECs analyzed by gelatin zymography.

## Discussion

Previous studies showed that PGAs induce expression of MMP-1, −2, −3, −9, and −17 and TIMP-1 and −2 in the human ciliary body [7, 10–15]. Here, we investigated the expression levels of MMPs and TIMPs in HNPCECs cultured with various concentrations of bimatoprost, latanoprost, or tafluprost, demonstrating different profiles with respect to increases in MMP and decrease in TIMP expression by each PGA.

Each PGA elicited a unique MMP and TIMP expression profile in HNPCECs. At a concentration of 1000 μM, bimatoprost induced greater expression of MMP-2 and MMP-3 than latanoprost and tafluprost. Bimatoprost has a greater effect on lowering IOP than latanoprost [16, 17]. The ability of PGAs to lower IOP may involve MMP-2 and −3. At a concentration of 1000 μM, tafluprost and latanoprost increased the expression MMP-1 and MMP-9, respectively, more than bimatoprost. Some reports showed no significant difference between bimatoprost and latanoprost [18, 19]. Furthermore, Alm showed no clinically significant difference in efficacy between latanoprost and tafluprost [18]. The MMP and TIMP expression profiles of each PGA may be different in individual patients. Thus, the decrease IOP by PGAs may be determined by several diverse factors.

The ratio of infiltration of each PGA from the ophthalmic solution into the aqueous humor and the concentration of each PGA may be essential for the efficacy of each agent. We investigated PGAs at 10, 100, and 1000 μM, concentrations intentionally ranged within those of commercial ophthalmic solutions. In clinical use, 0.03 % bimatoprost, 0.005 % latanoprost, and 0.0015 % tafluprost ophthalmic solutions are available, corresponding to 720 μM, 110 μM, and 33 μM, respectively. The concentration of each PGA in the aqueous humor is lower than that in the ophthalmic solution. In an in vitro study, peak aqueous concentrations after topical administration were 0.009 μg/ml, 0.028 μg/ml, and 0.00875 μg/ml, respectively [20–22], with each concentration corresponding to 22 nM, 65 nM, and 19 nM for bimatoprost, latanoprost, and tafluprost, respectively. The relative infiltration ratios of bimatoprost, latanoprost, and tafluprost from ophthalmic solutions into the aqueous humor are calculated as 1.00:19.3:18:8, respectively. This suggests a more favorable infiltration ratio of the ester prodrugs latanoprost and tafluprost than the free acid form of bimatoprost.

To measure the MMP and TIMP expression levels in HNPCECs in the present study, we did not use the ester prodrugs of latanoprost and tafluprost, but rather used the free acid forms to avoid differences in efficacy owing to hydrolysis by esterases. We obtained scattered MMP and TIMP expression levels when the ester forms of latanoprost and tafluprost were used in the experiments. The varying efficacy of PGAs due to hydrolysis by esterases in the cornea, depending on levels expressed in each patient, may consequently determine the effectiveness of latanoprost and tafluprost.

Our results showed a consistency in the expression levels of MMP-2 mRNA and MMP-2 enzyme activity as measured by qPCR and gelatin zymography, respectively. On the other hand, we did not detect other MMP bands by gelatin zymography, possibly because the levels were beyond the sensitivity of the assay. As a consequence, we estimated the enzymatic level of the other MMPs by the mRNA levels of each MMP.

Claudin-1 and occludin in HNPCECs and non-pigmented ciliary epithelial cells, which comprise the BAB in the uvea, are candidate substrates of MMPs. We previously showed that MMP-1, MMP-3, and MMP-9 degraded claudin-1 and occludin in HNPCECs and in non-pigmented ciliary epithelial cells of a swine ciliary body and showed increased expression of MMP-1, MMP-3, and MMP-9 in the presence of TNF-α in HNPCECs [9]. Interestingly MMP-17 can activate TNF-α [23]. These data suggest that inflammation in the ciliary body, like in iridocyclitis, may be involved in the reduction of IOP.

Fibrillin-1, versican, and hyaluronan (FiVerHy) in the ciliary nonpigmented epithelium are other candidate substrates of MMPs expressed in HNPCECs [24]. Versican was digested by MMP-3 [25], and fibrillin-1 was digested by MMP-2 and MMP-9 [26]. The increased expression levels of MMP-2, MMP-3, and MMP-9 in HNPCECs induced by PGAs may lead the degradation of the physiological complex of FiVerHy in the ciliary nonpigmented epithelium. The precise function of FiVerHy is still unknown, but the dense localization of hyaluronan in this high molecular complex on the surface of the ciliary nonpigmented epithelium may exhibit a protective effect of the ciliary nonpigmented epithelium from the MMPs in the vitreous humor [27–30], and the destruction of FiVerHy by MMPs may induce mild iridocyclitis.

## Conclusion

In conclusion, unique expression profiles of MMPs and TIMPs induced by bimatoprost, latanoprost, and tafluprost, as shown in HNPCECs, may contribute to clinically different effects on intraocular pressure decreases in patients with glaucoma or ocular hypertension. This information may be important in the selection of the best PGA for individual patients.

### Availability of materials and methods

The dataset supporting the conclusions of this article is available in the repository: https://mynotebook.labarchives.com/share_attachment/BLT%2520data/MTkuNXwxNjc1NTEvMTUtMi9UcmVlTm9kZS80MDg0NzExOTY0fDQ5LjU=

## Abbreviations

BAB: blood-aqueous barrier
DMSO: dimethyl sulfoxide
FBS: fetal bovine serum
FiVerHy: fibrillin-1, versican, and hyaluronan
HNPCEC: human non-pigmented ciliary epithelial cell
IOP: intraocular pressure
MMP: matrix metalloproteinase
PGA: prostaglandin analog
TIMP: tissue inhibitor of metalloproteinase
TNF: tumor necrosis factor
